# Capturing occupational risk of airborne disease: An international job-exposure matrix based on five exposure factors

**DOI:** 10.5271/sjweh.4235

**Published:** 2025-09-01

**Authors:** Karen M Oude Hengel, Susan Peters, Zara A Stokholm, Alex Burdorf, Anjoeka Pronk, Henrik A Kolstad, Martie van Tongeren, Ioannis Basinas, Vivi Schlünssen

**Affiliations:** 1 Netherlands Organization for Applied Scientific Research TNO, Unit Healthy Living and Work, Leiden, The Netherlands.; 2 Institute for Risk Assessment Sciences, Utrecht University, Utrecht, The Netherlands.; 3 Department of Occupational Medicine, Danish Ramazzini Center, Aarhus University Hospital, Aarhus, Denmark.; 4 Erasmus Medical Center Rotterdam, Department of Public Health, Rotterdam, The Netherlands.; 5 Centre for Occupational and Environmental Health, School of Health Sciences, Faculty of Biology, Medicine and Health, University of Manchester, Manchester, United Kingdom.; 6 Department of Public Health, Research Unit for Environment, Occupation and Health, Danish Ramazzini Centre, Aarhus University, Aarhus, Denmark.

**Keywords:** COVID-19, infectious disease, JEM, SARS-CoV-2 solar ultraviolet radiation

## Abstract

**Objective:**

This study aimed to construct a job-exposure matrix (JEM) for the risk of being infected by infectious agents through airborne or droplet transmission in an occupational setting, which might lead to a respiratory disease.

**Methods:**

An established COVID-19-JEM formed the basis for the development of the general airborne infectious agents JEM. Nine researchers in occupational epidemiology from three European countries (Denmark, The Netherlands and the United Kingdom) discussed and agreed on which factors from the COVID-19-JEM were relevant and whether new factors or adjustments of risk levels were needed. Adjustments to the COVID-19 JEM were made in a structured iterative. based on an expert assessment, a JEM on solar ultraviolet radiation (UVR) exposure including information on hours per day working inside, and national data on hours per week on site. Finally, a risk score was assigned to all factors for each job title within the International Standard Classification of Occupations system 2008 (ISCO-08).

**Results:**

This airborne infectious agents JEM contains five factors: (i) hours spent per week on site, (ii) hours spent per day working inside, (iii) number and (iv) nature of contacts, and (v) being in close physical contact to others. Per occupation, a risk score ranging from 1 (low risk) to 3 (high risk) was provided for all five factors separately.

**Conclusion:**

This newly developed infectious agents JEM assesses the risk at population level using five factors. Following validation, this JEM could serve as a valuable tool in future studies investigating the role of work in the occurrence of a respiratory disease.

During the COVID-19 pandemic, research showed that the workplace played an important role in the spread of SARS-CoV-2 (1). A worker could be exposed to SARS-Cov-2 from an infected coworker, patient or member of the general public, mainly through (i) inhalation of aerosols with the virus, (ii) deposition of droplets with the virus on eyes, nose, mouth or (iii) direct contact with infected individuals. Reviews showed that lack of ability to social distance (2), enclosed environments (3), and poor ventilation (4) were factors associated with a higher risk for a SARS-Cov-2 infection. Consequently, variation in SARS-Cov-2 infections across occupations was convincingly shown, for example among representative samples of workers in the United Kingdom (5) and The Netherlands (6). During the pandemic, European experts developed a job-exposure matrix (JEM) to assess the risk at job title level, using eight factors related to exposure to SARS-Cov-2 at the worksite (7).

Besides SARS-Cov-2, other infectious agents can also transmit from person to person through coughing, talking, and sneezing. Common diseases caused by infectious agents are influenza, common cold, chickenpox, and measles (8, 9). Working in close contact with individuals, particularly in confined spaces with lack of ventilation, increases the risk of transmission of these infectious agents (10). Current JEM on biological factors are scarce and include mainly animal-based exposures (11) or consider only healthcare workers (12). However, given that the process of airborne transmission is similar for SARS-CoV-2 and other airborne infections, the validated COVID-19-JEM will be a good bases for developing a JEM for exposure to other airborne infectious agents.

Because the COVID-19-JEM was developed during the pandemic – for the situation when general mitigation measures were in place to reduce transmission risk (ie, working from home, face covering in public places) – the existing JEM was adapted to account for the fact that these mitigation measures have been removed. The aim of the current study was to develop an airborne infectious agents JEM.

## Methods

The airborne infectious agents JEM was developed based on the existing COVID-19-JEM, which was supplemented with expert assessments, a solar ultraviolet radiation (UVR) exposure JEM, and national data on onsite working hours.

### COVID-19-JEM

The COVID-19-JEM was developed by applying a qualitative and iterative approach with expert meetings within and between countries (7). The COVID-19-JEM included four dimensions of transmission risk (number of contacts, nature of contacts, contaminated workspaces, and location), two mitigation measures (social distancing and use of face covering), and two precarious work dimensions (income insecurity and migrant workers). All 436 job titles within the International Standard Classification of Occupations from 2008 (ISCO-08) were assigned an exposure risk score (range 0–3) for each dimension.

### Development of the airborne infectious agents JEM

Nine experts from Denmark, The Netherlands, and the United Kingdom were involved. Two members (KOH and VS) drafted the first proposal for modifying the COVID-19-JEM. The revised JEM was finalized, including the definition of the risk scores and rules for assignment, in an iterative process.

Several adjustments in the factors were made to transform the COVID-19-JEM to an airborne infectious agents JEM. First, ‘use of face covering’ was removed because this measure has been largely abolished. ‘Contaminated workspaces’ was also removed as this transmission route, most importantly surface contamination, was less important than previously expected and we decided to focus on the airborne transmission route (13). Workers with precarious work have a higher risk for infections, such as SARS-Cov-2 (14). However, we decided to remove ‘precarious work’ as it not only relates to working conditions but living and housing conditions also play an important role. Second, definitions for ‘social distance’ and ‘location’ were modified. In the COVID-19-JEM, ‘social distance’ means the possibility to keep sufficient distance to others. Due to the non-pandemic situation, we changed the definition into “the extent to which a job requires close physical contact”. The factor ‘location’ was renamed into ‘hours per day working inside’ and adjusted to align with results from a recent JEM on solar UVR exposure (15). Finally, ‘hours per week working on site’ was added to the JEM, as the variation in number of hours on site differs large between occupations.

The COVID-19-JEM risk scores were also adapted. In the COVID-19-JEM, occupations consisting of homeworkers received a risk score of 0. As no occupations work exclusively from home nowadays, this risk score was removed in the airborne infectious JEM.

Additionally, country-specific ratings in the COVID-19-JEM were replaced by one global rating per factor and job title. Policies and measures taken by companies differed across countries during the pandemic, but these differences are minimal outside of a pandemic context.

### Assignment of risk scores

One expert from each country (SP, ZS, and IB) independently rated all risk scores for nature of contacts, number of contacts, and close physical contact for jobs where working from home was mandatory during the pandemic since the COVID-19-JEM did not contain ratings for these. All other risk scores of the COVID-19-JEM were also checked. The agreement of the individual ratings for nature and number of contacts and being in close physical contact with others were compared using two performance indicators [agreement score (AS) and weighted kappa (WK)]. Thereafter, the risk scores of the three experts were combined and compared. Differences were discussed and consensus was reached during a meeting. Overall rules on consensus per job title and factor were established *a priori*. Specifically, for differences in risk scores of one point, the majority rating was applied. For differences of ≥2 points, the majority rule was applied as default, but the score could be adapted based on discussion.

Two factors were based on external data sources. Risk scores for ‘hours per day working inside’ were retrieved from the JEM for solar UVR exposure (15). Data from The Netherlands Working Conditions Survey from November 2023 were used to calculate ‘the hours per week on site’ (16). When data was missing for a specific job title within this survey, the data were retrieved from a higher level within ISCO-08.

## Results

Descriptions of the five factors (‘hours per week on site’, ‘hours per day working inside’, ‘nature of contacts’, ‘number of contacts’, and ‘in close physical contact’) of the airborne infectious agents JEM are shown in table 1.

**Table 1:** The five factors, descriptions and risk categories ^a^ of airborne infectious agents job exposure matrix (JEM).

Factor	Description	Low risk (score=1)	Elevated risk (score=2)	High risk (score=3)
Hours per week on site	The number of working hours per week working outside the home environment.	Working ≤3 days on site (<24 hours)	Working 4 days per week on site (≥24 hours or <32 hours per week)	Working 5 days per week on site (≥32 hours)
Hours per day working indoors	The number of hours per day someone is working indoors.	0–4 hours/day	5–6 hours/day	≥7 hour/day
Number of contacts	The number of workers in close vicinity of each other	<10 per day	10–30 per day	>30 per day
Nature of contacts	Contacts with co-workers or general public (eg, clients, patients, students)	Working in workspaces with co-workers only	Working in workspaces with the general public	Working in workspaces with regular contacts in sectors with many airborne diseases
In close physical contact	The extent to which close ‘physical’ contact is required	Close ‘physical’ contact is not required	Close ‘physical’ contact is sometimes required	Close ‘physical’ contact is very (often) required

^a^ The factors, risk scores and the assignment were defined during expert meetings with 9 occupational epidemiologists from three European countries.

‘Hours working on site’ reflects the time a worker can be exposed at work to airborne infectious agents. This factor covers both the contract hours and the number of hours working from home as both largely vary across occupations. For example, more part-time workers are in female-dominated occupations such as teaching and nursing (17). Low, intermediate and high risk were respectively defined as working ≤3, 4 and 5 days per week on site.

‘Hours indoor working’ was included in the airborne infectious agents JEM because the risk of being exposed to an infectious agent is much higher when working indoors than outdoors (3). Low, intermediate and high risk were respectively defined as working ≤4, 5–6 and 7–8 hours/day inside.

The definition and risk scores for ‘number of contacts’ and ‘nature of contacts’ remained similar to the COVID-19-JEM. The factor ‘in close physical contact’ was refined into the extent to which close ‘physical’ contact is required. Low, intermediate and high risk were respectively defined as close ‘physical’ contact is not, sometimes and very often required.

Agreement between the three experts was good for number of contacts [AS: 0.84 (95% confidence interval (CI) 0.82–0.87) WK: 0.72 (95% CI 0.68–0.76)] and nature of contacts [AS: 0.88 (95% CI 0.86–0.90) WK: 0.78 (95% CI 0.75–0.82)], and moderate for in close physical contact [AS: 0.82 (95% CI 0.79–0.85) WK: 0.51 (95% CI 0.46–0.56)]. Consensus was reached for the risk scores for each factor per job title during discussions, whereby table 2 presents the risk scores for 10 job titles as examples. The final airborne infectious agents JEM can be found in supplementary file A (www.sjweh.fi/article/4235).

**Table 2:** Examples of ten job titles and their risk within each of the five factors of the airborne infectious agents job exposure matrix (JEM) ^a^.

Job title (ISCO-08)		Hours per week on site	Hours per day inside	Nature of contacts	Number of contacts	In close ‘physical’ contact to others
**No**	**Title**					
1211	Finance managers	2	3	1	1	1
2261	Dentists	2	3	3	2	3
3154	Air traffic controllers	3	3	1	1	1
3258	Ambulance workers	2	3	3	2	3
4120	Secretaries	1	3	1	1	1
5132	Bartenders	1	2	3	3	3
6114	Mixed crop growers	2	1	1	1	1
7115	Carpenters and joiners	3	2	2	1	2
8331	Bus and tram drivers	2	3	3	3	2
9123	Window cleaners	2	1	1	1	1

^a^ All job titles are presented in supplementary file A. All factors are assessed with a risk score to be exposed at the worksite (range 1–3), see table 1 for details.

## Discussion

The airborne infectious agents JEM contains five factors (‘hours per week on site’, ‘hours indoor working’, ‘number of contacts’, ‘nature of contacts’, ‘in close physical contact’). Each occupation within the ISCO-08 was assigned a risk score ranging from 1–3 for every factor, based on the COVID-19-JEM supplemented with an expert assessment, a JEM on solar UVR exposure and national data on hours per week on site.

A key strength of this study lies in the structured, iterative approach used to develop the general airborne infectious agents JEM. However, a notable limitation is that, by design, a JEM does not account for within-occupation variability. As a result, well-established risk factors such as inadequate ventilation and moisture (18) could not be incorporated, as their variability within occupations is likely to exceed the variability observed between occupations. Another limitation is that only Dutch data were used for the factor ‘hours per week on site’. However, we consider the data from this representative sample to be largely generalizable to other European countries. For occupations with missing data, we used an aggregated level of the ISCO-08, which led to a more crude estimation.

In line with the COVID-19-JEM (5, 19, 20), the first necessary step is the validation of the airborne infections agents JEM by estimating the associations of the factors with the prevalence of airborne infections (eg, influenza) from large observational studies (eg, register-based data). As the JEM is developed by one Nordic and two Western European countries, it is recommended to include also data from other European regions in the validity studies. To encourage other researchers to apply the airborne infectious agents JEM, this JEM is freely accessible in the supplementary file.

## Supplementary material

Supplementary material
